# Supplementary material for: Recent HIV infections among newly diagnosed individuals living with HIV in rural Lesotho: Secondary data from the VIBRA cluster-randomized trial
Source: PLoS One. 2022 Nov 21;17(11):e0277812. doi: 10.1371/journal.pone.0277812 (PMC9678280; doi:10.1371/journal.pone.0277812)
Supplement: Attachment — Submitted filename: response_to_reviewers.docx [file pone.0277812.s002.docx]

**Response to Reviewers**

*The recency of newly diagnosed HIV infections among the rural general population in Lesotho: Secondary data from the VIBRA cluster-randomized trial*

**Journal Requirements:**

*We have prepared our anonymized dataset and uploaded it onto Zenodo. The DOI is the following: 10.5281/zenodo.7230264*

*We included the DOI link in our data sharing statement.*

*Direct link:* [*https://zenodo.org/record/7230264#.Y1Fsj4LP3eo*](https://zenodo.org/record/7230264#.Y1Fsj4LP3eo)

**Reviewer #1:**

Thank you for the opportunity to review a revised version of the manuscript. It reads much better than the previous version. However there are a few more minor issues that need to be addressed

Abstract
1) Line 29-30: the figure 184 should appear in the results unless the study went out to purposively enroll 184

***Response:*** *We have adapted the abstract accordingly:*

Participants were recruited from August 2018 to May 2019 and 184 patient-samples included in this study.

2) Line 41-42: this sentence should say 7% of those who were newly diagnosed had acquired HIV in the preceding 6 months. It is difficult to follow as written at the moment

***Response:*** *We have adapted accordingly:*

During door-to-door testing among a general population sample in rural Lesotho, 7% of those who were newly diagnosed had acquired HIV in the preceding 6 months.

Introduction
3) Line 47- 48: should refer to targeted prevention interventions vs just targeted interventions

***Response:*** *Thank you for pointing this out. We rephrased the sentence:*

The identification of newly infected individuals is important to help HIV prevention programs to determine where interventions are most needed.

Results
4) Table 1: consider presenting viral loads on the log scale
***Response:*** *We changed the viral load to log scale.*

5) Table 2 : in the column on recent infections, for the categorical n/N (%) should be presented for both test and reference groups i.e. for Biological sex it should be 11/108 (10.2) for females vs 2/76 (2.6) for males.
for district it should be 1/16 (6.3%) for Butha Buthe vs 12/168 (7.1%) for Mokhotlong
please correct this for categorical variables

***Response:*** *Thank you for this important point. We adapted the table accordingly.*

**Reviewer #2:**

My comments were addressed. There is no more critique.

***Response:*** *Thank you for your thorough review.*
